# Case Report: Postoperative cervical lymph node metastasis of the neuroendocrine carcinoma component of rectal mixed adenoneuroendocrine carcinoma

**DOI:** 10.3389/fonc.2025.1464426

**Published:** 2025-08-20

**Authors:** Gao Quanwei, Yin Yiqiang, Wang Gangpu

**Affiliations:** Department of General Surgery, Fourth People’s Hospital of Jinan, Jinan, China

**Keywords:** case report, rectal mixed adenoneuroendocrine carcinoma, metastasis to cervical lymph nodes, component metastasis, corresponding treatment, prognosis

## Abstract

**Introduction:**

Clinical case reports of rectal mixed neuroendocrine-non-neuroendocrine tumors are rare. This report highlights a case in which only the neuroendocrine carcinoma component metastasized to the lymph nodes seven years postoperatively, and its successful treatment.

**Case description:**

A 73-year-old male was admitted to our hospital in November 2015 with rectal bleeding lasting more than four months. A mass was detected and radical surgery and preventive ileostomy was performed, followed by six cycles of chemotherapy. Postoperative pathology revealed two distinct histological patterns, representing the adenocarcinoma and neuroendocrine components; 6 of 12 mesenteric lymph nodes tested positive, whereas 2 pelvic lymph nodes were negative. The patient presented again on April 30, 2022, with multiple palpable masses of varying sizes in the right side of the neck. A biopsy revealed a metastatic poorly differentiated carcinoma consistent with neuroendocrine carcinoma in the right cervical lymph nodes, which was considered to originate from the rectum. The patient again underwent surgery and six rounds of chemotherapy, which resulted in a significant reduction in the size of the cervical lymph nodes.

**Discussion:**

Due to the highly malignant nature of mixed adenoneuroendocrine carcinoma, early diagnosis and treatment are crucial for improving patient survival and therapeutic outcomes. A comprehensive, individualized treatment plan involving surgery, chemotherapy, targeted therapy, and immunotherapy can provide better patient outcomes. Given the metastatic potential of mixed adenoneuroendocrine carcinoma, long-term postoperative follow-up is essential.

## 1 Introduction

Recent years have seen an estimated ten-fold increase in the incidence of rectal mixed neuroendocrine-non-neuroendocrine tumors (1, 2). However, their incidence remains rare compared with other rectal malignancies. Mixed adenoneuroendocrine carcinoma (MANEC), a subtype of mixed neuroendocrine-non-neuroendocrine tumors, is characterized by the presence of both adenocarcinoma and neuroendocrine carcinoma (NEC) components, each constituting at least 30% of the tumor (3, 4). MANEC is highly aggressive and associated with poor prognosis, with the median survival time of patients with metastatic MANEC ranging from one month to a maximum of only 12–19 months (5). Early diagnosis and treatment are crucial for improving patient survival rates. The detection of tumor markers, as a non-invasive diagnostic tool, is crucial for the early diagnosis of MANEC and the dynamic monitoring of its progression. Commonly used tumor markers include carcinoembryonic antigen, cancer antigen (CA)19-9, and CA72-4. These markers enable clinicians to assess disease status, monitor recurrence, and detect metastasis.

Surgical resection remains the preferred treatment for patients with rectal MANEC, with the extent and approach dependent on the tumor size, location, depth of invasion, and presence of metastasis. Radical surgery is the primary option for localized lesions, and may involve radical resection or local excision. In patients with tumors <1 cm in size without muscularis propria invasion, endoscopic treatment or local excision can be considered. For tumors >1 cm in size, surgical resection should be approached with greater caution. In cases in which lymph node metastasis or invasion of the intestinal wall has occurred, more extensive radical surgeries should be considered (7). Given the aggressiveness of the NEC component of MANEC, postoperative adjuvant chemotherapy is critical. Platinum-based chemotherapy is widely used in patients with MANEC owing to its efficacy against NEC. Etoposide + cisplatin is currently the first-line chemotherapy protocol and is particularly effective for neuroendocrine tumors with a Ki-67 index of >55% (8). Second-line regimens such as carboplatin + etoposide or fluorouracil + oxaliplatin + leucovorin may be considered for patients who develop resistance to platinum-based chemotherapy, and have demonstrated efficacy in certain cases. Targeted therapy and immunotherapy have also emerged as novel treatment options for MANEC. In patients with high microsatellite instability or mismatch repair deficiency, treatment with immune checkpoint inhibitors may significantly improve prognosis. Studies also indicate that combining anti-angiogenic therapy, such as bevacizumab, can prolong progression-free and overall survival, particularly in tumors with a NEC component. These emerging therapies provide potential personalized treatment options for refractory or recurrent MANEC, further advancing the development of comprehensive treatment strategies.

## 2 Case presentation

### 2.1 Patient information

A 73-year-old male was admitted to the hospital on November 12, 2015, presenting with hematochezia lasting over four months, accompanied by poor nutritional status, progressive weight loss, and difficulty defecating. The patient had no significant history of underlying diseases or major surgeries. He had a 40-year history of smoking, occasional alcohol consumption, no known drug allergies, and was not on long-term medication. There was no family history of cancer.

### 2.2 Clinical findings

Upon admission, routine blood tests, coagulation assessments, tumor marker tests, and erythrocyte sedimentation rate measurements were within normal ranges. However, imaging revealed a suspected mass at the rectosigmoid junction with possible pelvic lymph node metastasis.

**Figure 1:**
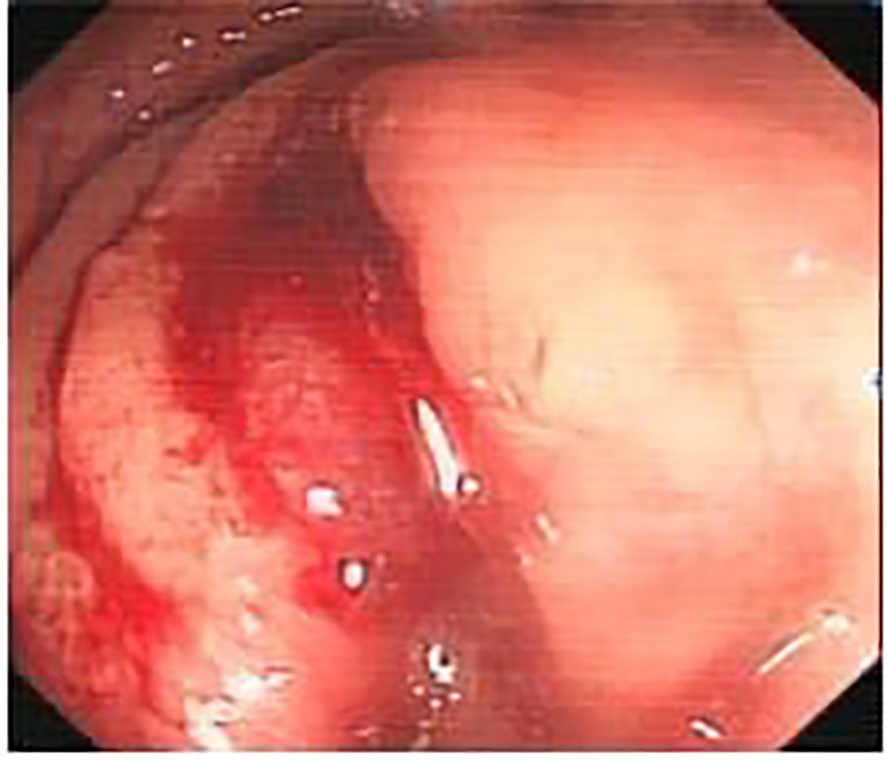
The tumor, indicated by the green arrow, can be seen growing intraluminally, with a surface characterized by hyperemia and edema, and prone to bleeding.

A colonoscopy performed on November 18, 2015, identified a circumferential mass approximately 12 cm from the anal verge, with surface congestion, edema, erosion, and significant luminal narrowing (**Figure 1**).

### 2.3 Diagnostic assessment

**Figure 2:**
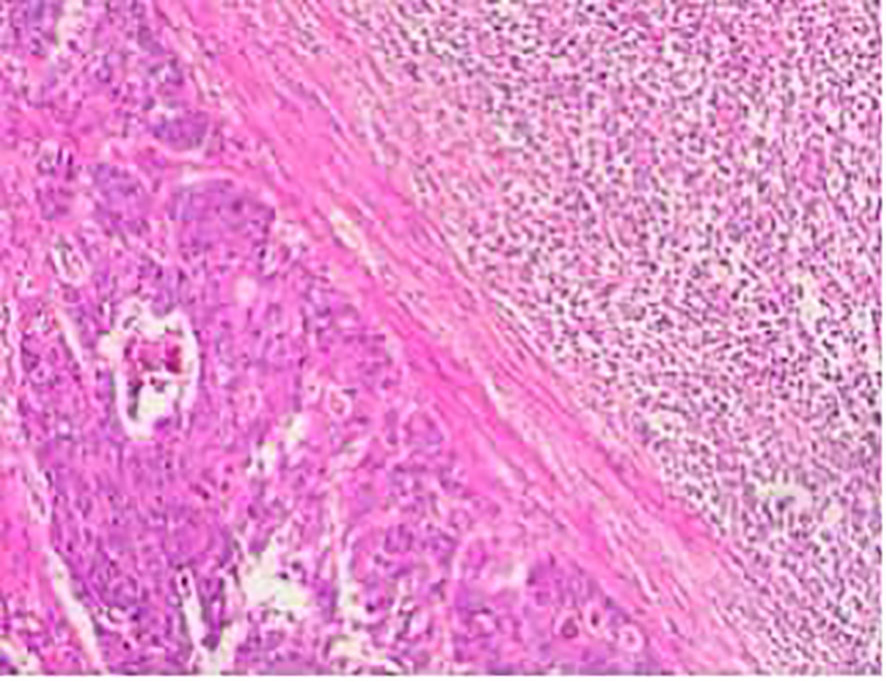
Tumor cells exhibiting small, uniform nuclei with fine cytoplasm. Chromogranin A staining was negative in the adenocarcinoma region (bottom left image).

The patient was diagnosed with mixed adenoneuroendocrine carcinoma (MANEC) of the rectum, a rare and aggressive malignancy containing both adenocarcinoma and neuroendocrine carcinoma (NEC) components. Differential diagnoses included conventional adenocarcinoma, squamous cell carcinoma, and gastrointestinal stromal tumors (GIST). Postoperative histopathological and immunohistochemical analysis confirmed the diagnosis, revealing a poorly differentiated adenocarcinoma component (CK8/18(+), CDX2(+), C-erB-2(3+)) and a highly aggressive NEC component (Synaptophysin(+), CD56(+), Ki-67 (~40%)), indicating significant proliferative activity. Additional markers showed CK(+), chromogranin A(-), and LCA(-). These findings confirmed the biphasic nature of the tumor, with a dominant high-grade NEC component, necessitating an aggressive treatment approach (**Figure 2**).

## 3 Therapeutic intervention

The patient underwent radical rectal resection with preventive ileostomy on November 27, 2015. Postoperative pathology confirmed a 5.8 × 3.5 cm tumor, with muscularis propria and adipose tissue invasion, localized perineural invasion, and intravascular tumor thrombi. Six out of twelve mesenteric lymph nodes tested positive for metastasis, while two pelvic lymph nodes were negative. Following surgery, the patient received six cycles of adjuvant chemotherapy (oxaliplatin 150 mg + capecitabine 60 mg). The ileostomy was closed six months postoperatively, and the patient was placed on routine follow-up.

## 4 Follow-up and outcomes

**Figure 3:**
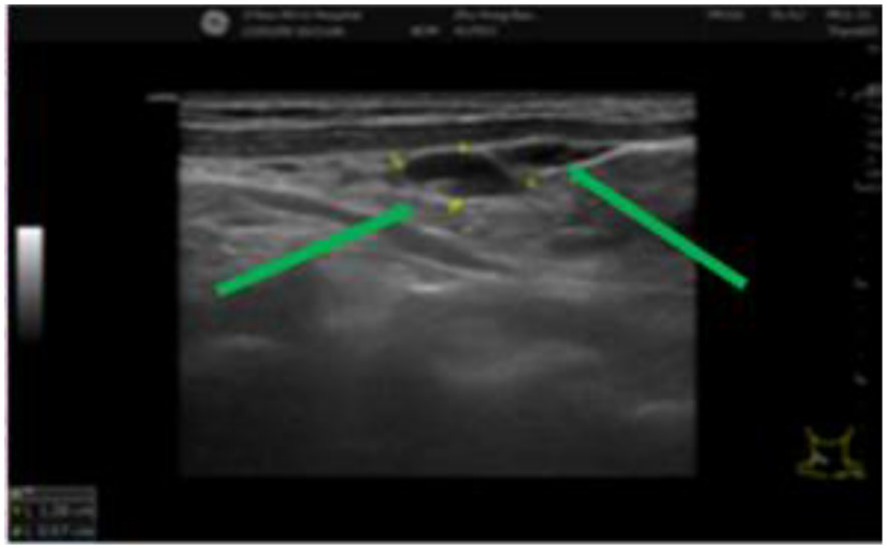
Multiple cervical lymph nodes of varying sizes can be seen and are indicated by green arrows. The lymph nodes exhibit clear structures and well-defined boundaries.

**Figure 4:**
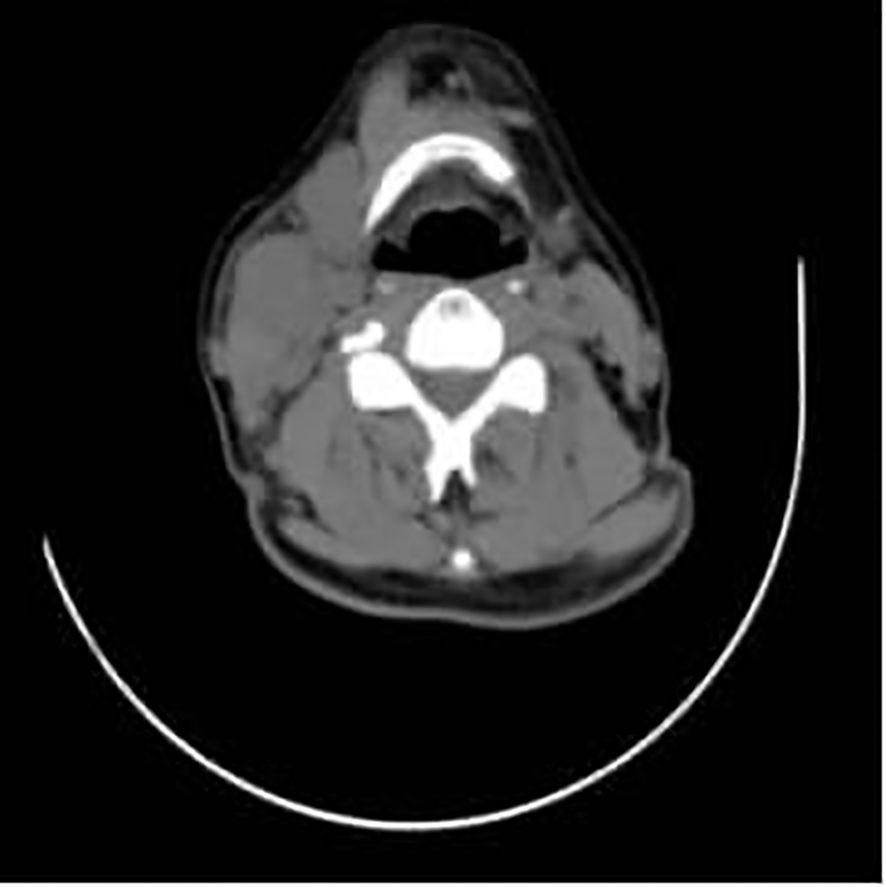
A mass can be seen in the right cervical region, with surrounding tissues compressed and displaced, and is indicated by the red arrow.

**Figure 5:**
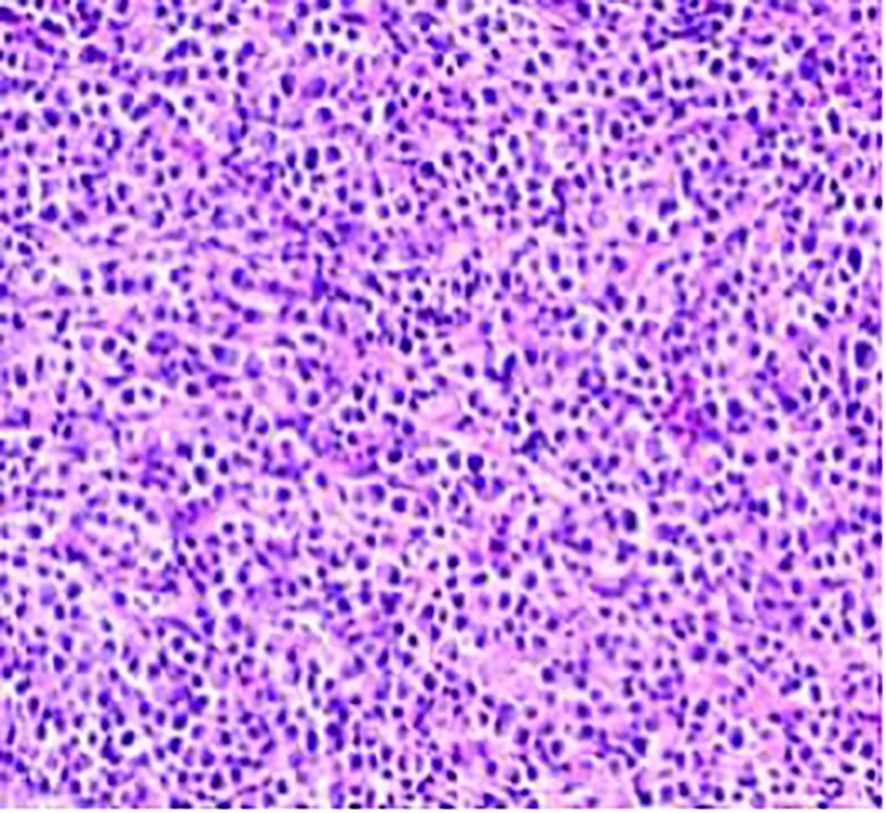
The neuroendocrine carcinoma component, consisting of small, uniform cells with a high nucleus-to-cytoplasm ratio and frequent mitotic figures.

**Figure 6:**
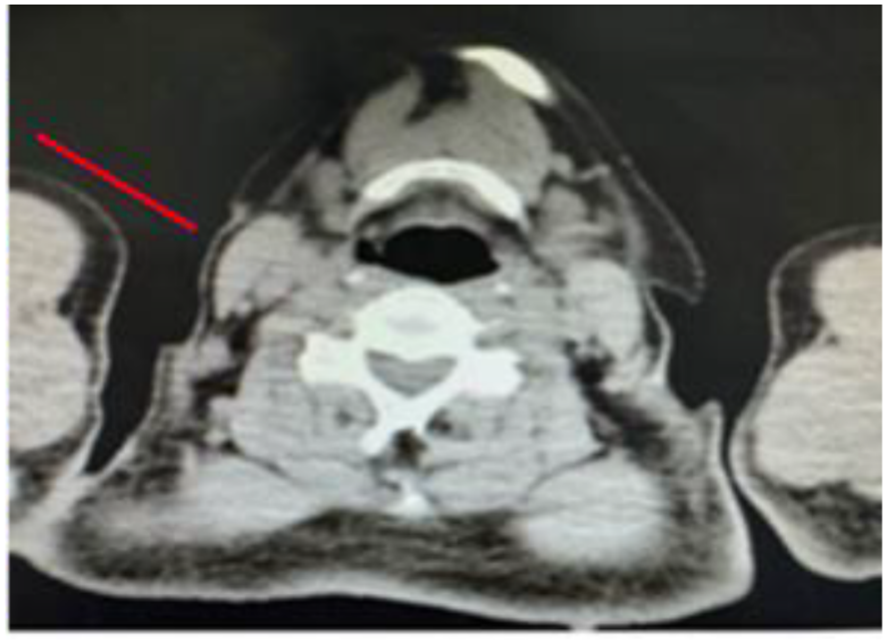
Cervical lymph nodes adjacent to the surgical site, indicated by the red arrow, are significantly reduced in size, with local tissue structural disorder. There are no obvious signs of systemic metastasis.

During a five-year follow-up period (2015–2020), routine whole-body CT scans showed no recurrence or metastasis, and tumor markers (AFP, CEA, CA19-9) remained within normal limits, except for CA72–4 fluctuations (6.39–14.33 U/mL). However, on April 30, 2022, the patient developed multiple enlarging lumps in the right cervical region. Imaging confirmed multiple enlarged cervical lymph nodes (**Figures 3**, **4**), with elevated CA19-9 (90.70 U/mL) and CA72-4 (20.00 U/mL). A biopsy on May 11, 2022, confirmed poorly differentiated metastatic neuroendocrine carcinoma (NEC), originating from the rectal tumor. Immunohistochemistry revealed positive staining for CK, CK20, CDX-2, Villin, synaptophysin, and CD56, with a high Ki-67 index (~40%), suggesting aggressive disease (**Figure 5**). The patient underwent six cycles of etoposide + cisplatin chemotherapy, leading to significant shrinkage of cervical lymph nodes, and follow-up CT (**Figure 6**) scans showed no further metastasis or recurrence. You can clearly see the main treatment process and follow-up content of the patient from **Table 1**.

**Table 1:** Timeline of treatment and follow-up.

Date	Event
11-12-2015	Patient admitted with hematochezia for more than 4 months. Initial blood tests and imaging studies performed. CT suggested a mass at the rectosigmoid junction with lymph node metastasis.
11-18-2015	Colonoscopy revealed a circumferential mass located 12 cm from the anal verge, with luminal narrowing. Biopsy confirmed adenocarcinoma.
11-27-2015	Radical rectal cancer surgery with preventive ileostomy performed. Postoperative pathology confirmed mixed adenoneuroendocrine carcinoma with mesenteric lymph node metastasis (6/12). Postoperative chemotherapy with six cycles of oxaliplatin (150 mg) and capecitabine (60 mg) was initiated.
05-25-2016	First follow-up: Whole-body CT showed no signs of recurrence or metastasis. Tumor marker levels: AFP, 3.1 ng/mL; CEA, 6.5 ng/mL; CA19-9, 18.2 U/mL; CA72-4, 7.02 U/mL. Ileostomy closure surgery performed.
11-25-2016	Second follow-up: Whole-body CT showed no signs of recurrence or metastasis. Tumor marker levels: AFP, 5.6 ng/mL; CEA, 7.8 ng/mL; CA19-9, 11.4 U/mL; CA72-4, 6.39 U/mL.
05-24-2017	Third follow-up: Whole-body CT showed no signs of recurrence or metastasis. Tumor marker levels: AFP, 7.2 ng/mL; CEA, 5.3 ng/mL; CA19-9, 22.6 U/mL; CA72-4, 9.12 U/mL.
11-20-2017	Fourth follow-up: Whole-body CT showed no signs of recurrence or metastasis. Tumor marker levels: AFP, 4.4 ng/mL; CEA, 8.1 ng/mL; CA19-9, 15.7 U/mL; CA72-4, 10.04 U/mL.
11-15-2018	Fifth follow-up: Whole-body CT showed no signs of metastasis. Tumor marker levels: AFP, 6.3 ng/mL; CEA, 9.2 ng/mL; CA19-9, 24.9 U/mL; CA72-4, 9.90 U/mL.
11-10-2019	Sixth follow-up: Whole-body CT showed no signs of recurrence or metastasis. Tumor marker levels: AFP, 2.9 ng/mL; CEA, 4.6 ng/mL; CA19-9, 16.3 U/mL; CA72-4, 12.75 U/mL.
05-08-2020	Seventh follow-up: Whole-body CT showed no signs of recurrence or metastasis. Tumor marker levels: AFP, 8.5 ng/mL; CEA, 3.9 ng/mL; CA19-9, 14.5 U/mL; CA72-4, 14.33 U/mL.
04-30-2022	Multiple masses in the right cervical region reported by the patient. CT and ultrasound confirmed lymph node enlargement. Tumor marker levels: CA19-9, 90.70 U/mL; CA72-4, 20.00 U/mL.
05-11-2022	Right cervical lymph node biopsy confirmed metastatic poorly differentiated neuroendocrine carcinoma. Immunohistochemistry: CK20(+), CDX-2(+),synaptophysin(+), Ki-67(~40%).
05–2022 to 01-2023	Six cycles of cisplatin 40 mg + etoposide 100 mg chemotherapy administered. Follow-up CT showed significant shrinkage of the cervical lesions and no further metastasis.

The normal reference ranges for the laboratory markers in the table are as follows: AFP (0–10 ng/mL), CEA (0–5 ng/mL for non-smokers, 0–10 ng/mL for smokers), CA19-9 (0–37 U/mL), and CA72-4 (0–6 U/mL).AFP, alpha-fetoprotein; CA, cancer antigen; CEA, carcinoembryonic antigen; CT, computed tomography.

## 5 Discussion

In the case reported here, fluctuations in CA72–4 levels during the postoperative follow-up period prompted the performance of additional imaging assessments, ultimately confirming NEC metastasis. The dynamic monitoring of tumor marker levels not only indicates potential recurrence risk but also aids in guiding treatment decisions; this tool is thus critical for the management of MANEC. Postoperative immunohistochemical analysis in this case revealed distinct adenocarcinoma and neuroendocrine components. Positive markers for the adenocarcinoma component included CK8/18, CDX2, and C-erB-2, whereas positive markers for the neuroendocrine component included chromogranin A, synaptophysin, CD56, and a high Ki-67 index (~40%). Ki-67 is a key prognostic marker, as patients with a Ki-67 index ≥55% have a mortality risk eight-fold higher than those with a Ki-67 index ≤55%. The early detection of changes in tumor marker levels and comprehensive immunohistochemical profiling can provide critical insights into the nature and prognosis of MANEC, aiding in more precise patient management and better outcomes.

The patient described in this report underwent radical rectal cancer surgery in 2015, and postoperative pathology revealed mesenteric lymph node metastasis of only the adenocarcinoma component. More than seven years later, cervical lymph node metastasis of the NEC component occurred. The mechanisms underlying this metastasis remain unclear but may involve several factors. First, the NEC component of MANEC is characterized by a higher Ki-67 proliferation index than that of the adenocarcinoma component, indicating its strong proliferative and invasive potential (6, 9, 10). A study by Wang et al. (11) reported that the vascular endothelial growth factor (VEGF) positivity rate was significantly higher in tumors with high Ki-67 expression than in those with low Ki-67 expression. VEGF, the most potent proangiogenic factor (12), promotes endothelial cell proliferation and tumor angiogenesis by acting on specific tyrosine kinase receptors and VEGF receptors 1 and 2, facilitating tumor growth. VEGF also enhances vascular permeability within tumors, promoting the invasion and metastasis of NEC cells. Tumor neovascularization mediated by VEGF often results in fragile structures, such as a single layer of endothelial cells with weak or absent smooth muscle and basement membranes, enabling easier penetration by tumor cells (13). This may explain why the NEC component is more prone to distant metastasis via vascular and lymphatic routes, while the adenocarcinoma component is often confined to the primary site or regional lymph nodes. Another significant factor is the neuroendocrine-specific marker chromogranin A, which has been shown to promote the proliferation of NEC cells and modulate the tumor microenvironment by inhibiting fibroblast adhesion within the tumor (14, 15). Moreover, in the NEC tumor microenvironment, stromal cells express abundant platelet-derived growth factor (PDGF). PDGF, a growth factor secreted by various cells, including platelets, macrophages, endothelial cells, and epithelial cells, activates multiple signaling pathways in other malignancies, promoting tumor cell proliferation, intratumoral angiogenesis, inhibition of apoptosis, chemoresistance, and tumor progression. However, the relationship between NEC differentiation and proliferation and PDGF and PDGF receptor expression within the tumor stroma remains unclear (16). Finally, more infiltrating lymphocytes are often observed in metastatic NEC than in primary lesions. These infiltrates include CD3+CD4+ T cells, CD3+CD8+ T cells, and often regulatory T cells. Regulatory T cells suppress antitumor immune responses within the tumor microenvironment by inhibiting the functions of effector T cells, B cells, and natural killer cells, thereby weakening the antitumor immune response and promoting tumor growth and progression (17). In summary, the aggressive and metastatic characteristics of the NEC component of MANEC may be closely related to its high proliferative capacity, angiogenesis, regulation of the tumor microenvironment, and immune evasion. These factors may have collectively contributed to the distant metastasis of the NEC component of MANEC in the case reported here.

This case highlights the importance of proactive follow-up strategies. A follow-up period of up to 10 years should be incorporated into the treatment plan, particularly for patients with NEC components. Vigilance is necessary to detect potential NEC metastases, even in cases with no recurrence for many years postoperatively. Fluctuations in tumor marker levels should prompt further imaging investigations to detect early signs of recurrence or metastasis.

Given the highly malignant nature of MANEC, early diagnosis and treatment are crucial to improve patient survival and therapeutic outcomes. A comprehensive and individualized treatment plan that integrates surgery, chemotherapy, targeted therapy, and immunotherapy may provide better therapeutic outcomes. Prolonged surveillance and a multidisciplinary approach are essential to optimize outcomes and address the unique challenges associated with MANEC management.

## 6 Extended literature review and discussion

In recent years, studies investigating the relationship between lymph node status and prognosis in visceral organ tumors have gradually increased. For instance, in the field of pancreatic ductal adenocarcinoma, a prospective lymphadenectomy protocol study demonstrated that the specific anatomical locations of lymph node metastases (such as stations 13, 14, and the mesenteric lymph nodes of the small intestine) have independent prognostic significance for tumor staging, recurrence risk, and overall survival (18). This study confirmed that dissection of the first-echelon lymph nodes is sufficient for accurate staging, whereas metastases in certain specific stations (for example, involvement of station 14 and the small intestinal mesenteric lymph nodes) serve as important indicators of poorer prognosis. Based on these findings, we propose that, in cases of rectal mixed adenoneuroendocrine carcinoma (MANEC), attention should also be paid to the impact of lymph node metastasis location on patient prognosis and treatment decision-making. To further clarify the prognostic significance of different lymph node stations in MANEC, future research should increase sample size and employ multicenter, prospective study designs to explore the relationship between various metastasis sites and patient survival as well as treatment response. This approach will not only help optimize the current staging system but also provide a scientific basis for developing more effective comprehensive treatment strategies.

## Data Availability

The original contributions presented in the study are included in the article/Supplementary Material. Further inquiries can be directed to the corresponding author.
